# A novel herbal formula, SGE, induces endoplasmic reticulum stress-mediated cancer cell death and alleviates cachexia symptoms induced by colon-26 adenocarcinoma

**DOI:** 10.18632/oncotarget.24616

**Published:** 2018-03-27

**Authors:** Aeyung Kim, Minju Im, Jin Yeul Ma

**Affiliations:** ^1^ Korean Medicine (KM) Application Center, Korea Institute of Oriental Medicine (KIOM), Dong-gu, Daegu 701-300, Republic of Korea

**Keywords:** colon cancer, cachexia, weight loss, muscle wasting, herbal medicine

## Abstract

Cachexia in cancer patients, characterized by marked involuntary weight loss and impaired physical function, is associated with a poor prognosis in response to conventional treatment and with an increase in cancer-related mortality. Prevention of skeletal muscle loss under cancer-induced cachexia via inhibition of pro-cachectic factors, as well as a reduction in tumor mass, has been considered reasonable pharmacological and nutritional interventions to treat cancer patients. In this study, we constructed a novel herbal formula, SGE, which contains *Ginseng Radix alba, Atractylodis Rhizoma alba*, and *Hoelen*, examined its anti-cancer and anti-cachexia efficacies. In *in vitro* experiments, SGE induced death of CT-26 murine colon carcinoma cells via endoplasmic reticulum stress, and suppressed the production of inflammatory cytokines in Raw 264.7 murine macrophage-like cells. In addition, SGE treatment attenuated CT-26-induced C2C12 skeletal muscle cell atrophy as well as CT-26-induced reduction in lipid accumulation in 3T3-L1 adipocyte. In CT-26 tumor-bearing mice, daily oral administration of 10 and 50 mg/kg SGE remarkably attenuated the cachexia-related symptoms, including body weight and muscle loss, compared with saline treatment, while food intake was not affected. These data collectively suggest that SGE is beneficial as an anti-cancer adjuvant to treat cancer patients with severe weight loss.

## INTRODUCTION

Colorectal cancer (CRC) is the third most commonly diagnosed cancer and the second leading cause of cancer-related deaths in adults in the U.S [1, 2]. In 2017, the American Society of Cancer estimated 95,520 new cases of colon cancer and 39,910 new cases of rectal cancer diagnosed in the U.S [3]. As the tumor grows, CRC patients demonstrate various symptoms, such as rectal bleeding, persistent abdominal discomfort, changes in bowel habits including constipation or diarrhea, weakness or fatigue, and decreased appetite [4]. Particularly, more than 50% of CRC patients experience unintentional weight loss with severe muscle wasting, and this condition, which is distinct from starvation and age-related muscle loss, cannot be prevented or recovered by conventional nutritional support [5, 6]. In cancer patients, wasting syndrome, including anorexia, metabolic and endocrine alterations, fatigue, and loss of lean body mass, is known as cancer cachexia, and this condition diminishes the efficacy of conventional chemotherapy and radiotherapy, reduces quality of life, and worsens the prognosis of cancer patients. In fact, at least 20% of cancer patients die from cachectic symptoms such as weight loss [7–9]. Therefore, to overcome colon cancer, it is necessary to suppress cancer cells directly and control cancer-induced cachectic symptoms, particularly for preservation of lean body mass.

In recent years, pathophysiological mechanisms underlying the wasting of skeletal muscle and adipose tissue during cancer cachexia have been investigated intensely, and key processes and therapeutic targets have been identified, including pro-inflammatory mediators (e.g., IL-6, TNF-α, IL-6, IL-1, and IFN-γ) and proteolysis mediators (e.g., myostatin, proteolysis-inducing factor, angiotensin II, and the ubiquitin–proteasome system) secreted from the tumor cell themselves and host cells in response to tumors [8, 10, 11]. Several medicinal trials to control cancer cachexia have been performed using synthetic drugs that stimulate the appetite, suppress the inflammatory response, reduce proteolysis, and increase protein synthesis [12–14]; however, their use is limited because of their unexpected side effects and low *in vivo* efficacy. In addition, antibodies or synthetic peptides targeting cachectic mediators have been effective in reversing cachexia conditions [15, 16]; however, these agents have a high cost and lack of clinical data for their effectiveness as well as safety. Recently, herbal medicines have proven to be beneficial for managing cancer-induced cachexia symptoms, including anorexia, weight loss, fatigue, and muscle wasting, in tumor-bearing mice because of their multi-modal pharmacological actions and low toxicity [17–19].

In this study, we formulated a novel herbal cocktail, SGE, which is composed of *Ginseng Radix alba, Atractylodis Rhizoma alba,* and *Hoelen*. *Ginseng Radix alba*, unprocessed ginseng root called white ginseng, has been used for thousands of years as a tonic to elevate mood and reduce fatigue and has been reported to have anti-diabetic, anti-hypertensive, anti-hyperglycemic, anti-depressant, and hemopoietic effects [20–22]. *Atractylodis Rhizoma alba* is a commonly used medicinal herb with anti-inflammatory, anti-osteoporotic, anti-cancer, and anti-melanogenic activities [23–25]. *Hoelen* is a subterranean mushroom that grows on the roots of pine trees and has long been used as a diuretic, sedative, and remedy for gastric diseases in Eastern traditional medicine [26]. Despite their many pharmacological properties, the efficacies of these components against cancer-induced cachexia, either alone or in combination as an herbal cocktail, have not been demonstrated.

In the present study, we examined whether SGE suppresses tumor growth and alleviates cachexia symptoms in mice bearing CT-26 colon carcinomas. Furthermore, we elucidated the anti-cancer and anti-cachectic mechanisms in detail using murine CT-26 colon carcinoma cells, Raw 264.7 macrophage-like cells, C2C12 myoblasts, and 3T3-L1 adipocytes.

## RESULTS

### SGE inhibits proliferation and induces apoptotic cell death in CT-26 murine colon carcinoma cells

**Figure 1:**
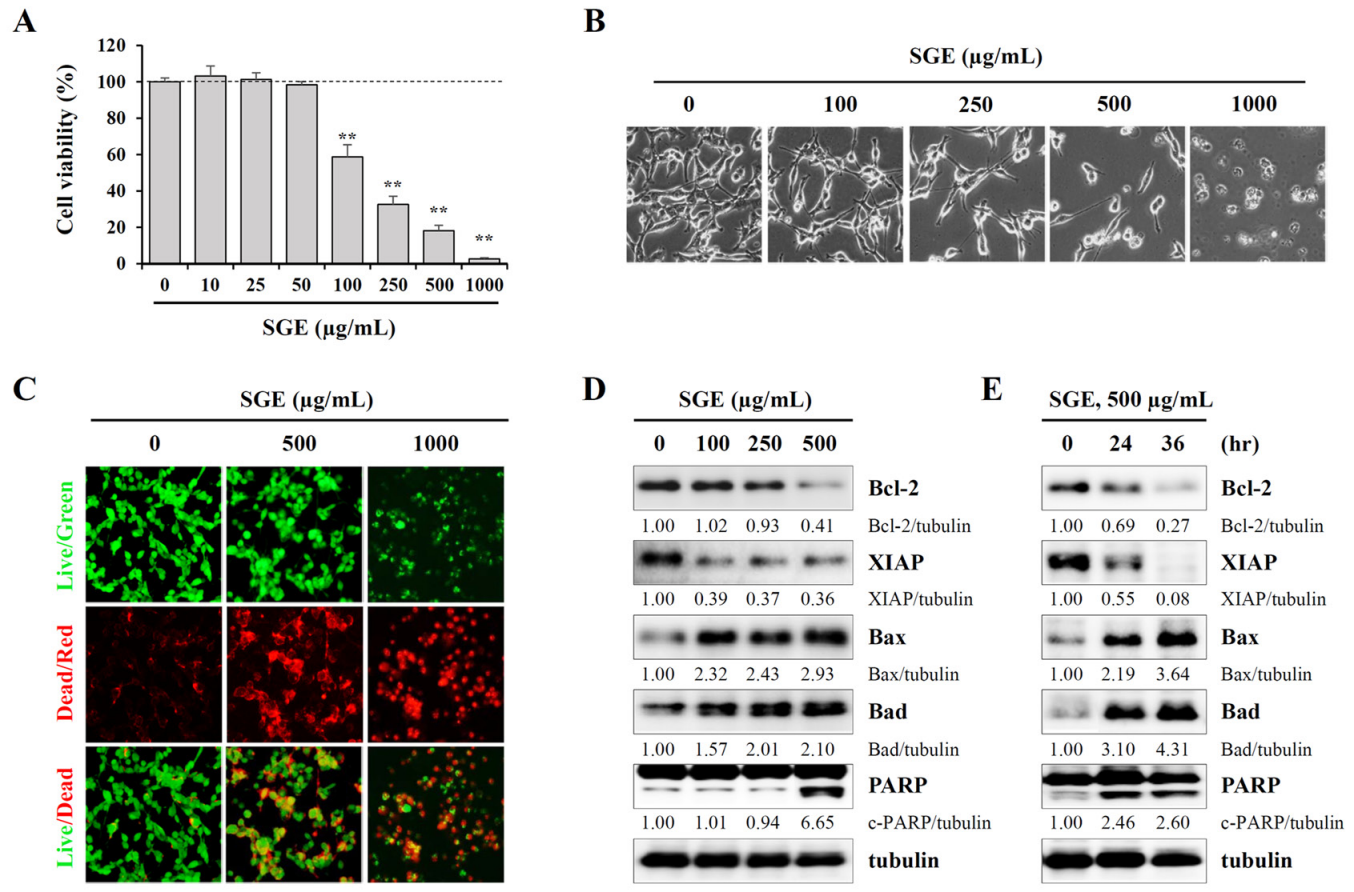
SGE decreases viability and induces apoptotic death in CT-26 murine colon carcinoma cells **(A)** CT-26 cells seeded in 96-well culture plates were incubated with SGE (10–1000 μg/mL) for 48 h, and then cell viability was measured using the CCK-8 kit. The data are representative of three independent experiments performed in triplicate and are expressed as means ± SD. ^**^p < 0.01 vs. untreated control. **(B)** The morphological changes in SGE-treated CT-26 cells were observed under an inverted microscope at ×200 magnification. **(C)** CT-26 cellsplated on 12-well culture plates were incubated with SGE (0, 500, 1000 μg/mL) for 36 h. After labeling cells using the LIVE/DEAD Cell Imaging Kit, live (green) and dead (red) cells were observed under a fluorescence microscope. **(D-E)** The levels of cell death-related proteins were analyzed by Western blotting in cells treated with the indicated concentrations of SGE for 24 h (D) or in cells treated with 500 μg/mL SGE for 24 and 36 h (E). The relative band intensities were calculated using ImageJ software after normalizing to tubulin expression.

To examine whether SGE can affect the proliferation and viability of CT-26 cells, we measured viable cells by the CCK-8 assay after treating cells with increasing concentrations of SGE for 24 h. As shown in Figure 1A and 1B, SGE inhibited cell proliferation and induced severe cytotoxicity in a dose-dependent manner at concentrations of 100 μg/mL or higher, and the morphology of the cells was almost completely collapsed at a concentration of 1000 μg/mL (F=339.4, *p* < 0.0001, one-way ANOVA). In the LIVE/DEAD cell imaging assay, SGE treatment induced a significant decrease in green fluorescent live cells and a concomitant increase in red fluorescent dead cells (Figure 1C). Western blotting showed that SGE remarkably down-regulated the levels of anti-apoptotic proteins, including Bcl-2 and XIAP, and up-regulated the levels of pro-apoptotic proteins, including Bax, Bad, and cleaved PARP, in dose- and time-dependent manners (Figure 1D and 1E). Because SGE is an herbal mixture consisting of three herbs *Ginseng Radix alba*, *Atractylodis Rhizoma alba*, and *Hoelen*, we next examined the effects of ethanol extracts of each herb on cell viability. As shown in Supplementary Figure 1, treatment with ethanol extracts of each herb and co-treatment with all three herbs up to 500 μg/mL did not induce cytotoxicity, indicating that these herbs exert greater anti-proliferative activity when used together in an herbal cocktail.

### SGE induces phosphorylation of MAPK and AMPK, as well as ER stress, in CT-26 murine colon carcinoma cells

**Figure 2:**
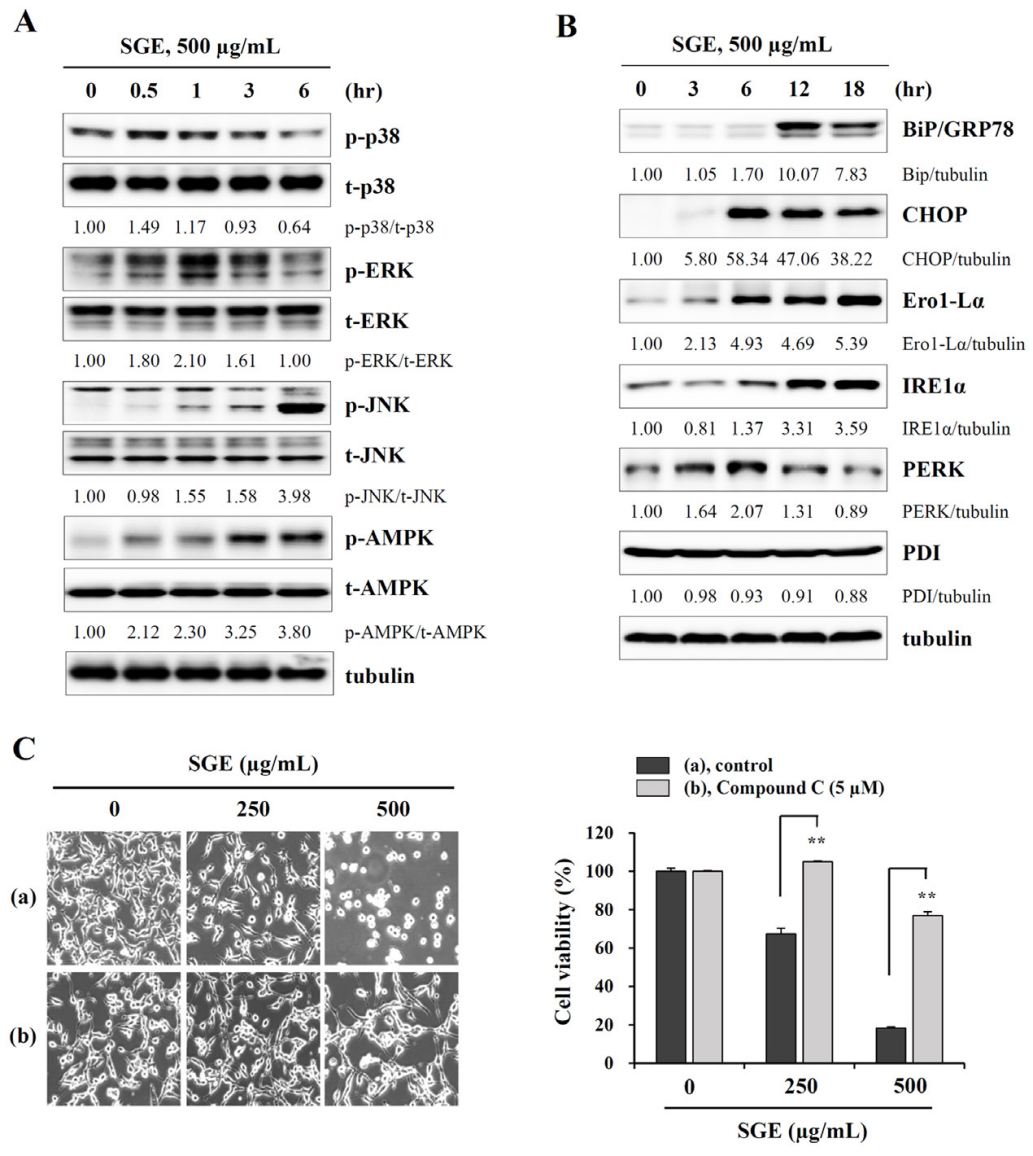
SGE increases the phosphorylation of MAPK and AMPK and induces ER stress **(A)** CT-26 cells were treated with 500 μg/mL SGE for 0.5, 1, 3, and 6 h, and the levels of total and phosphorylated p38, ERK, JNK, and AMPK were examined by Western blotting. **(B)** The levels of ER stress-related proteins were measured by Western blotting in CT-26 cells after treating with 500 μg/mL SGE for 3, 6, 12, and 18 h. The data are representative of three independent experiments, and the relative band intensities were calculated using ImageJ software after normalizing to tubulin expression. **(C)** Cells pretreated with or without compound C (5 μM) for 1 h were treated with 250 and 500 μg/mL SGE. After incubation for 24 h, cell viability was assessed by the CCK assay, and cell morphology was observed under an inverted microscope. ^**^p < 0.01 vs. untreated control.

It has been reported that prolonged ER stress can trigger cell death due to an impaired unfolded protein response [27], and MAPK activation has been implicated in ER stress-induced cell death [28]. In addition, AMPK which comprises a catalytic α-subunit and two regulatory subunits (β and γ) is activated under metabolic stress, ultimately inducing cell death [29]. As shown in Figure 2A, Western blotting revealed that SGE treatment rapidly increased the levels of phosphorylated p38 and ERK at 30 min post-treatment, and gradually decreased these levels after 1 h. Meanwhile, SGE also induced phosphorylation of JNK and AMPK, up to 24 h. In addition, ER stress-related proteins, including Bip, CHOP, Ero1-Lα, IRE1α, and PERK, were remarkably increased by SGE, but PDI was not affected (Figure 2B). To investigate the role of MAPK and AMPK activation in SGE-mediated cell death, CT-26 cells were pre-treated with pharmacological inhibitors of p38 (SB203580), ERK (PD98059), JNK (SP600125), and AMPK (compound C) before SGE treatment. As shown in Figure 2C and Supplementary Figure 2, pre-treatment with compound C effectively protected CT-26 cells from SGE-mediated cell death to approximately 80% at 500 μg/mL SGE, whereas PD98059 and SP600125 induced weak protection and SB203580 little effect. These data indicate that AMPK activation followed by ER stress is crucial for SGE-mediated cell death in CT-26 cells.

### SGE suppresses LPS-induced production of inflammatory cytokines and NO, expression of iNOS, and activation of MAPK and NF-κB in murine macrophages

**Figure 3:**
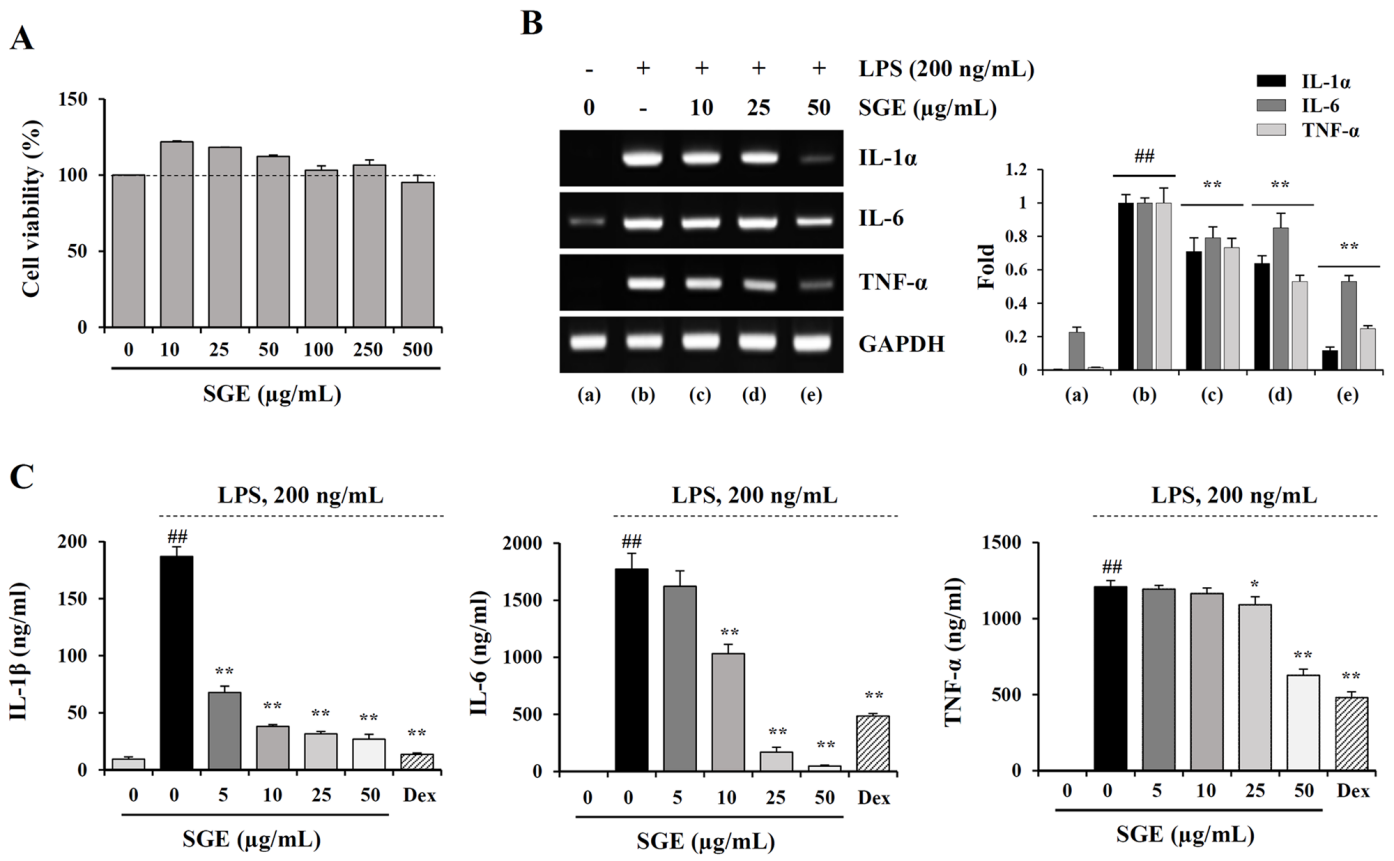
SGE inhibits LPS-induced production of inflammatory cytokines in murine peritoneal macrophages **(A)** After seeding peritoneal macrophages on 96-well culture plates, SGE (10–500 μg/mL) was added to the wells and incubated for 24 h. Cell viability was determined using the CCK-8 kit. The data are expressed as means ± SD performed in triplicate. **(B)** Cells pretreated with or without 10, 25, and 50 μg/mL SGE for 1 h were stimulated with LPS (200 ng/mL) for 6 h, and the mRNA levels of IL-1α, IL-6, and TNF-α were determined by RT-PCR. The band intensities relative to those of SGE-untreated cells were normalized to GAPDH and expressed as means ± SD from two independent experiments. **(C)** Cells were pretreated with or without 5, 10, 25, and 50 μg/mL SGE or dexamethasone (10 μM) for 1 h and stimulated with LPS (200 ng/mL) for 24 h. After collecting the culture supernatants, the levels of IL-1β, IL-6, and TNF-α were measured by ELISA and expressed as means ± SD performed in triplicate. ^#^*p* < 0.01 vs. untreated control, ^**^p < 0.01 vs. SGE-untreated control.

**Figure 4:**
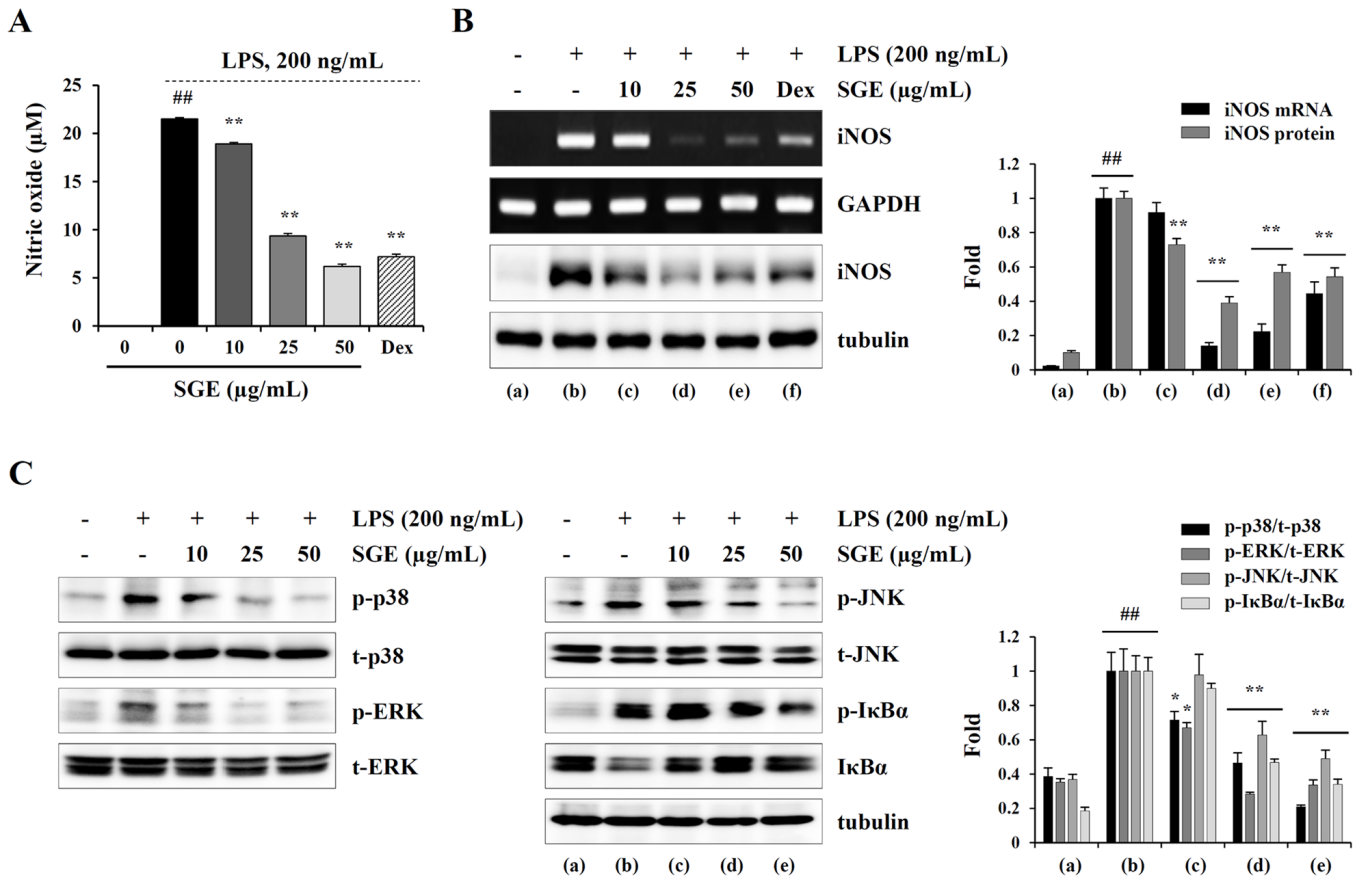
SGE inhibits LPS-induced NO production and MAPK/NF-κB activation in Raw 264.7 cells **(A)** Cells were pretreated with or without the indicated concentrations of SGE or dexamethasone (10 μM) for 1 h and then stimulated with LPS (200 ng/mL). After 24 h, the levels of NO in the culture supernatants were measured. The data are representative of three independent experiments and expressed as means ± SD performed in triplicate. **(B)** The mRNA and protein levels of iNOS in cells treated as described in (A) were examined by RT-PCR and Western blotting, respectively. The data were expressed as means ± SD of two independent experiments. **(C)** Cells pretreated with or without the indicated concentrations of SGE for 1 h were stimulated with LPS (200 ng/mL) for 1 h and then subjected to Western blotting. The band intensities relative to the SGE-untreated cells were determined and represented as means ± SD from two independent experiments. ^#^*p* < 0.01 vs. untreated control, ^*^p < 0.05 and ^**^p < 0.01 vs. SGE-untreated control.

It has been demonstrated that the chronic inflammatory response in cancer patients can elicit and accelerate cancer-induced cachexia symptoms, including anorexia, weight loss, nausea, lipolysis, and muscle wasting [8, 30]. Therefore, restraining the inflammatory response is considered a very effective anti-cachectic strategy. We next examined whether SGE can inhibit the production of pro-inflammatory and pro-cachectic cytokines such as IL-1, IL-6, and TNF-α in LPS-stimulated macrophages. As shown in Figure 3A, SGE at concentrations up to 100 μg/mL did not induce cytotoxicity in murine peritoneal macrophages; thus, we treated macrophages with 5, 10, 25, and 50 μg/mL SGE in this study. The mRNA levels of IL-1α, IL-6, and TNF-α were significantly increased after LPS stimulation, whereas they were considerably decreased by SGE pretreatment in a dose-dependent manner (IL-1; F=6.574e+008, *p* < 0.0001, IL-6; F=1.198e+008, *p* < 0.0001, TNF-α; F=4.45e+008, *p* < 0.0001, one-way ANOVA) (Figure 3B). In culture supernatants, the secreted levels of IL-1β, IL-6, and TNF-α was also remarkably decreased in SGE-treated cells compared with untreated control cells, similar to the effects of dexamethasone, used as the positive control (IL-1; F=539.3, *p* < 0.0001, IL-6; F=206.3, *p* < 0.0001, TNF-α; F=111.6, *p* < 0.0001, one-way ANOVA) (Figure 3C). Additionally, in Raw 264.7 cells, SGE efficiently reduced LPS-induced NO production and iNOS expression at both the mRNA and protein levels to extents comparable to those induced by dexamethasone (NO production; F=2926, *p* < 0.0001, iNOS mRNA; F=258.0, *p* < 0.0001, iNOS protein; F=132.6, *p* < 0.0001, one-way ANOVA) (Figure 4A and 4B). As reported in previous studies, production of inflammatory cytokines was considerably inhibited by treatment with ethanol extract with single herb, but the efficacy of SGE was superior to that of single herb. In particular, the efficacy of each herb at 16.7 μg/mL, that is concentration corresponding to SGE 50 μg/mL, was insignificant, indicating that these herbs have greater anti-inflammatory activity when used as an herbal cocktail (Supplementary Figure 3). It has been demonstrated that MAPK and NF-κB activation is critical for the LPS-induced production of pro-inflammatory cytokines. We found that LPS stimulation significantly increased the levels of phosphorylated p38, ERK, and JNK in Raw 264.7 cells, while SGE pre-treatment efficiently decreased their levels in a dose-dependent manner. In addition, LPS-induced phosphorylation and degradation of IκBα were also remarkably decreased in SGE-treated cells (p-p38; F=75.28, *p* < 0.0001, p-ERK; F=70.46, *p* < 0.0001, p-JNK; F=34.05, *p* < 0.0001, p-IκBα/IκBα; F=144.7, *p* < 0.0001, one-way ANOVA) (Figure 4C).

### SGE attenuates CT-26 CM-mediated inhibition of C2C12 myoblast proliferation and CT-26 CM-mediated C2C12 myotube wasting

**Figure 5:**
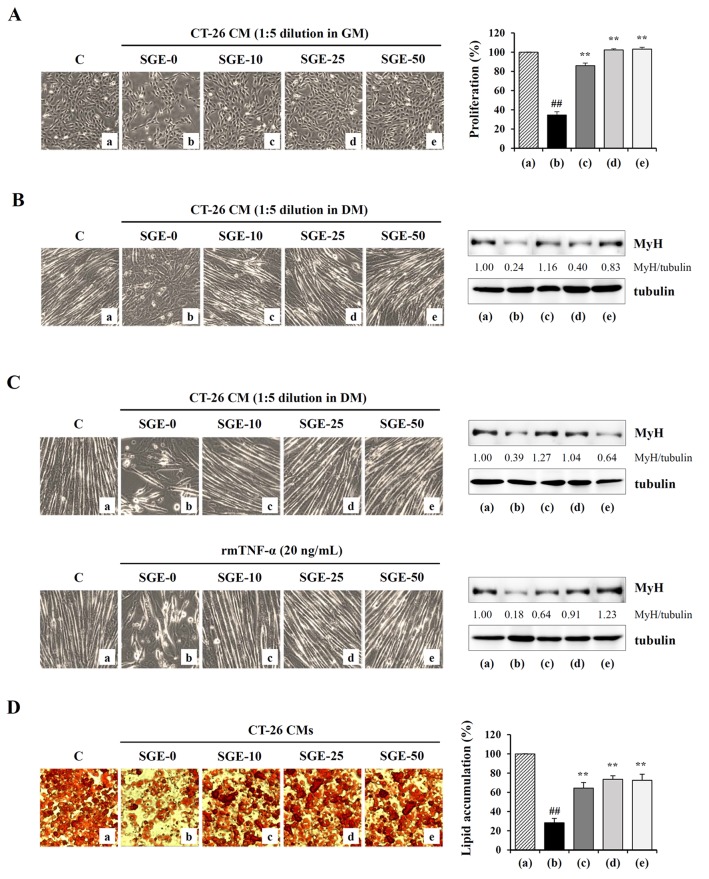
SGE attenuates CT-26 CM-induced muscle atrophy in C2C12 cells and lipolysis of 3T3-L1 adipocytes **(A)** The C2C12 myoblasts were treated with SGE-treated or -untreated CT-26 CM after dilution with GM and incubated for 48 h. Cells were observed under an inverted microscope, and viable cells were measured by the CCK-8 assay. The data are expressed as means ± SD performed in triplicate. **(B)** To induce myogenic differentiation, the C2C12 myoblasts were incubated in DM or in SGE-treated or -untreated CT-26 CM after dilution with DM. After 3 days, cells were observed under an inverted microscope, and the expression of MyH was detected by Western blotting. **(C)** The C2C12 myotubes differentiated in DM for 3 days were further incubated in DM or in SGE-treated or -untreated CT-26 CM for 48 h. Additionally, the C2C12 myotubes were treated with TNF-α (20 ng/mL) for 48 h in the presence or absence of SGE. Cells were observed, and MyH expression was determined by Western blotting. The relative band intensities were calculated using ImageJ software after normalizing to tubulin. **(D)** Mature 3T3-L1 adipocytes were treated with SGE-treated or untreated CT-26 CM for 48 h, and lipid accumulation in cells was measured by Oil Red O staining. The data are representative of three independent experiments and expressed as means ± SD performed in triplicate. ^#^*p* < 0.01 vs. CT-26 CM untreated control, ^**^p < 0.01 vs. SGE-untreated control.

In previous studies, treatment with CT-26 CM suppressed myoblast proliferation and differentiation into myotubes and accelerated myotube degradation, suggesting that tumor-derived factors such as myostatin and IL-6 promote skeletal muscle wasting [31, 32]. To examine the effects of SGE on tumor-induced muscle wasting, we first measured C2C12 myoblast proliferation after treatment with SGE-treated or -untreated CT-26 CM diluted 1:5 in GM for 48 h. As shown in Figure 5A, SGE-untreated CT-26 CM severely suppressed C2C12 myoblast proliferation by approximately 65% compared with control GM, whereas treatment with 10, 25, and 50 μg/mL SGE-treated CT-26 CM did not significantly affect C2C12 myoblast proliferation (F=523.4, *p* < 0.0001, one-way ANOVA). Particularly, cells incubated in 25 and 50 μg/mL SGE-treated CM were affected similarly to those incubated in control GM. Next, we examined whether SGE attenuates CT-26 CM-mediated muscle atrophy by inhibiting C2C12 myoblast differentiation into myotubes and degrading C2C12 myotubes. Similar to previous studies, we observed that SGE-untreated control CT-26 CM significantly impaired C2C12 differentiation compared with the control DM, accompanied by reduced myotube numbers and MyH expression. Meanwhile, SGE-treated CT-26 CM did not severely prevent C2C12 myoblast differentiation, exhibiting more increased myotube formation and MyH expression compared with CT-26 control CM (Figure 5B). When differentiated C2C12 myotubes were treated with CT-26 CM or TNF-α, they were remarkably degraded in morphology, and the levels of MyH in the myotubes were reduced. However, SGE-treated CT-26 CM or SGE treatment prior to TNF-α stimulation almost completely prevented myotube degradation and the decreased MyH expression (Figure 5C). Additionally, the effect of SGE on CT-26 CM-mediated lipolysis in well-differentiated adipocytes was evaluated. As shown in Figure 5D, the incubation of 3T3-L1 adipocytes with CT-26 CM decreased lipid accumulation to approximately 28% of lipid levels in 3T3-L1 adipocytes treated with fresh culture medium, whereas SGE-treated CT-26 CM decreased lipid accumulation to approximately 65–73%, suggesting that SGE inhibits CT-26 CM-mediated lipolysis (F=50.17, *p* < 0.0001, one-way ANOVA).

### Daily oral administration of SGE in CT-26 tumor-bearing mice alleviated weight loss and suppressed tumor growth

**Figure 6:**
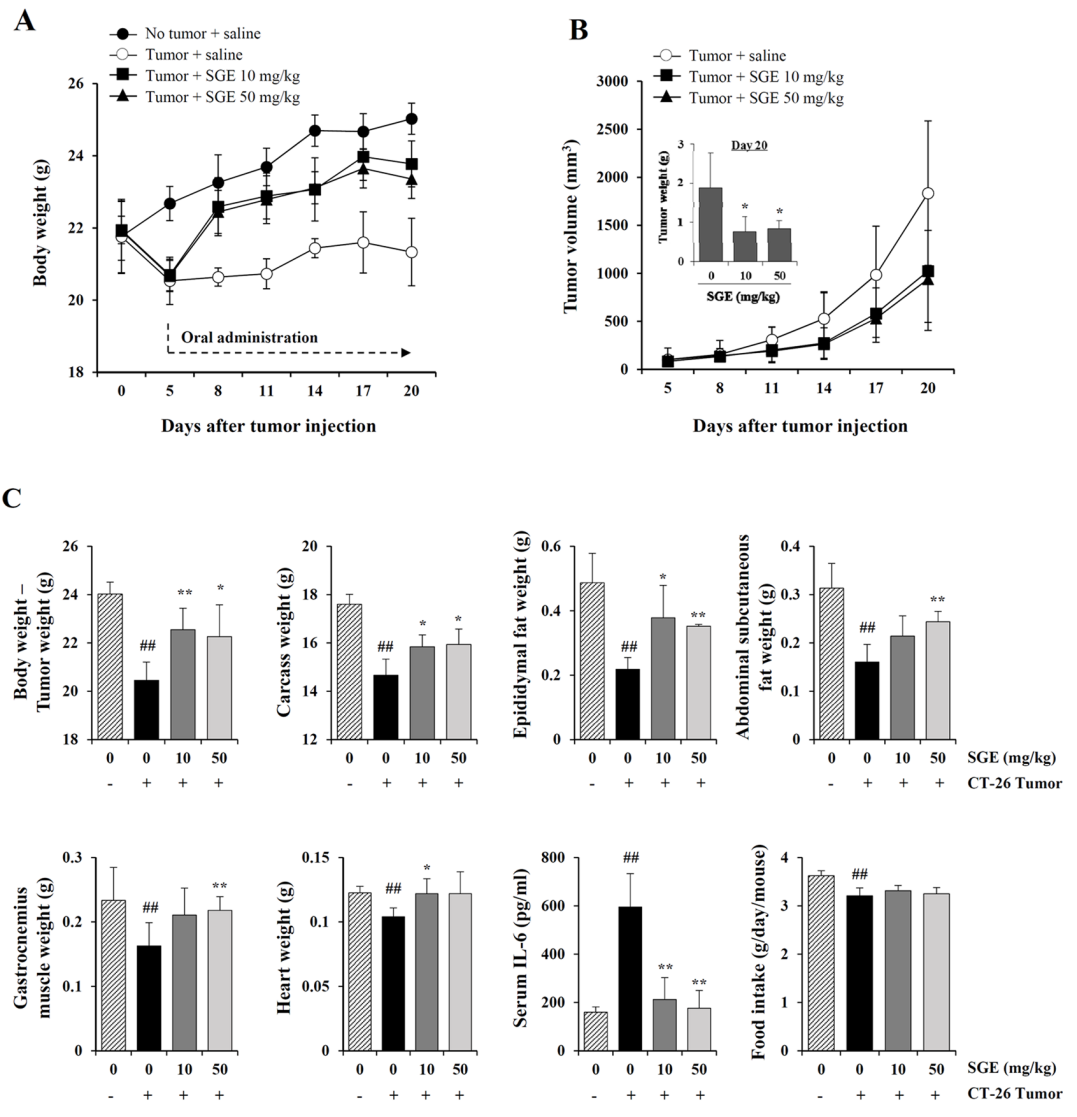
SGE administration alleviates weight loss and retards tumor growth in CT-26 tumor-bearing mice **(A–B)** Male BALB/c mice (n=15) were subcutaneously inoculated with CT-26 cells (3×10^6^/mouse). After 5 days, tumor-bearing mice were randomly divided into three groups (n=5 per group) and were administered saline or SGE daily at doses of 10 and 50 mg/kg for 15 days. Age-matched normal mice with no tumors (n=5) were also administered an equal volume of saline daily during the experiment. The body weight and tumor volume were measured every 3 days and expressed as means ± SD for each group. ^*^p < 0.05 vs. saline-treated control. **(C)** On day 20, the mice were sacrificed, the tumors excised, and the carcass, epididymal fat, abdominal subcutaneous fat, gastrocnemius muscle, and heart weighed. The IL-6 levels in the sera were determined by ELISA. The data are representative of three independent experiments and expressed as means ± SD of each group. ^#^*p* < 0.01 vs. normal group, ^*^p < 0.05 and ^**^p < 0.01 vs. saline-treated control.

In *in vitro* experiments, SGE induced CT-26 cell death and inhibited CT-26-mediated muscle wasting, lipolysis, and the LPS-induced inflammatory response. Therefore, we next examined the *in vivo* efficacy of SGE treatment in terms of its anti-cancer and anti-cachectic effects in CT-26 tumor-bearing mice. As shown in Figure 6A, the body weight of normal healthy mice steadily increased during the experimental period, whereas the CT-26 tumor burden decreased body weight by approximately 5.7% at 5 days after tumor injection; the difference in body weight between normal and tumor-bearing mice was approximately 10% regardless of the tumor weight. On the other hand, body weight was significantly increased in CT-26 tumor-bearing mice treated with 10 and 50 mg/kg SGE, showing a recovery of approximately 95% and 93.3% of the body weight of normal mice, respectively (on day 20). Consistent with the inhibitory effect of SGE on CT-26 cell proliferation *in vitro*, we observed that 10 and 50 mg/kg SGE administration significantly suppressed tumor growth by 44.2% and 48.8%, respectively, in CT-26 tumor-bearing mice compared with the saline-treated control mice on day 20 (Figure 6B). Control mice had a mean tumor weight of 1.88±0.89 g, while 10 and 50 mg/kg SGE-treated mice had mean tumor weights of 0.79±0.36 g and 0.84±0.20 g, showing 57.98% and 55.32% suppression, respectively (F=5.881, *p* = 0.0166, one-way ANOVA). As shown in Figure 6C, at the time of sacrifice, cachexia-related parameters, including the weights of the final body mass, carcass, epididymal fat, abdominal subcutaneous fat, gastrocnemius muscle, and heart, as well as the average food intake/day/mouse, were significantly reduced in saline-treated control mice compared with those in normal mice. In addition, the serum IL-6 level was dramatically elevated in saline-treated CT-26 tumor-bearing mice. By contrast, oral administration of 10 and 50 mg/kg SGE considerably prevented the losses in final body weight (F=6.270, *p* = 0.0137), carcass weight (F=6.862, *p* = 0.0103), and heart weight (F=3.452, *p* = 0.0654), wasting of adipose tissue (F=9.562, *p* = 0.0033) and skeletal muscle (F=8.001, *p* = 0.0062), and elevation of serum IL-6 (F=24.70, *p* < 0.0062) in CT-26 tumor-bearing mice, while SGE treatment did not restore appetite. These results collectively indicate that SGE reduced tumor burden, suppressed inflammatory responses, and alleviated CT-26 tumor-induced cachexia symptoms.

## DISCUSSION

The word “Cachexia” comes from the Greek words kakos, meaning bad, and hexia, meaning condition, and describes a multifactorial wasting syndrome characterized by excessive weight loss and depletion of fat and skeletal muscle. Cachexia occurs in 10–40% of patients with chronic illnesses, including renal and liver failure, chronic obstructive pulmonary disease, acquired immune deficiency syndrome, rheumatoid arthritis and cancer, and affects more than 5 million people in the U.S [5, 33]. Particularly, cancer-induced cachexia is one of the most critical factors accounting for the high morbidity and mortality in up to 80% of patients with advanced cancer, and almost 20% of cancer patients die directly from cachexia-induced weight loss. In addition, cancer-induced cachexia causes poor responses to conventional chemotherapy and radiotherapy in terms of efficacy and adverse effects and decreases survival and quality of life [34, 35]. Several agents exhibit considerable effectiveness in managing cancer cachexia by stimulating appetite (e.g., megesterol acetate, cannabinoids, corticosteroids, and ghrelin) and regulating pro-cachectic mediators (e.g., non-steroidal anti-inflammatory drugs, eicosapentaenoic acid, and β-hydroxy-β-methylbutyrate) [7]. However, these drugs have shown limitations in terms of their low bioavailability and unwanted side effects, and there have been no approved treatments for cachexia until now.

There is considerable evidence showing that the reversal of weight and muscle mass loss is the therapeutic goal for cancer cachexia rather than the stimulation of appetite [36–38]. Recently, several herbal medicines, including Hochuekkito (TJ-41) and *Coptidis rhizoma*, have been demonstrated to attenuate cancer cachexia by reducing IL-6 production, which is a critical mediator of muscle wasting [39, 40]. In addition, ZBHP, comprising the herbs *Rhizoma Anemarrheana* and *Cortex Phellodendri*, increased body weight and suppressed tumor-induced muscle atrophy by inhibiting pro-inflammatory cytokines and muscle catabolism in a C26 model [19]. In our previous studies, *Citrus unshiu* peel extract and the Oriental traditional herbal formula Sosiho-tang (Xiaochaihu-tang in Chinese, Sho-saiko-to in Japanese) efficiently improved cachexia-related symptoms in CT-26 tumor-bearing mice by reducing systemic inflammation and muscle degradation [32, 41]. In some cases, the symptoms of cachexia have been alleviated by inhibiting tumor growth, stimulating appetite, and removing tumor-derived pro-cachectic factors such as IL-6 and myostatin [42]. Because cancer cachexia is a complex syndrome that involves various host- and tumor-derived factors, multidisciplinary herbal medicines may be a good remedy for its treatment.

In the current study, we formulated an herbal mixture called SGE, composed of *Ginseng Radix alba*, *Atractylodis Rhizoma alba*, and *Hoelen*, based on the anti-cancer, anti-inflammatory, anti-fatigue, and immune-enhancing effects of each herb. We found that SGE at a concentration greater than 100 μg/mL efficiently induced CT-26 cell death by regulating the expression of anti- and pro-apoptotic proteins and ER stress induction, and AMPK activation was essential for SGE-mediated cell death (Figure 1 and 2). Interestingly, SGE exhibited more potent anti-proliferative activity as an herbal mixture compared with each herb alone or their co-treatment (Supplementary Figure 1). In addition, SGE remarkably inhibited the production of pro-inflammatory cytokines via suppression of iNOS expression and MAPK/NF-κB activation in Raw 264.7 cells (Figure 3 and 4). Moreover, SGE-treated CT-26 CM did not severely impair C2C12 myoblast proliferation or differentiation, but it efficiently prevented C2C12 myotube degradation, to a similar extent as that induced by normal GM or DM. In addition, SGE-treated CT-26 CM showed little effect on lipid accumulation in 3T3-L1 adipocytes (Figure 5). As demonstrated in *in vitro* experiments in which SGE possesses anti-cancer, anti-inflammatory, anti-muscle wasting, and anti-lipolysis activities, SGE administration in CT-26 tumor-bearing mice resulted in considerable recovery of body weight and significant suppression of tumor growth compared with saline treatment (Figure 6A and 6B). Skeletal muscle mass, fat mass, and heart weight decreased by CT-26 burden were also increased by SGE administration, while food intake was similar between saline- and SGE-treated mice. In addition, the serum IL-6 level elevated by tumor burden was also dramatically reduced by SGE administration, supporting the beneficial effects of SGE on cancer-induced cachexia symptoms (Figure 6C). On the other hand, administration of SGE to healthy normal mice without tumors showed no significant effect of increasing body weight compared to the control group administered with saline (Supplementary Table 2). In addition, SGE administration had little effect on the weight changes of major organs and parameters associated with hepatic and renal toxicity, reinforcing the efficacy of SGE to alleviate cancer-induced weight loss with no adverse effects (Supplementary Tables 3 and 4).

In addition to cachexia induced by cancer itself, anti-cancer agents also cause cachexia symptoms accompanied by weight and muscle loss, which can affect the efficacy of the agents and the survival rate of the patients. Cisplatin is a chemotherapeutic agent widely used for the treatment of several solid tumors. Recently, cisplatin reportedly activated the p38/CEBP-β and proteome/autophagy system, increased myostatin and inflammatory cytokines, and decreased Akt and myogenin/myoD, ultimately leading to increased proteolysis, decreased muscle mass and strength, and weight loss [43, 44]. Therefore, agents that mitigate the cisplatin-induced cachexia symptoms might be applied to chemotherapy as anti-cancer adjuvants. In this regard, herbal medicines that have multimodal pharmacological activities may be useful to overcome these conditions, and research on the preventive and therapeutic efficacy of SGE against cisplatin-induced cachexia is warranted.

In summary, the present study demonstrated that SGE reduces tumor mass, systemic inflammation, and cachexia symptoms in CT-26 tumor-bearing mice, followed by prevention of cancer-induced muscle and fat degradation. These data indicate that SGE is an effective novel herbal treatment for cancer patients when combined with anti-cancer chemotherapeutic agents.

## MATERIALS AND METHODS

### Cell lines

CT-26 murine colon carcinoma cells (American Type Culture Collection [ATCC] CRL-2638), Raw 264.7 murine macrophage-like cells (ATCC TIB-71), C2C12 murine myoblasts (ATCC CRL-1772), and 3T3-L1 murine pre-adipocytes (ATCC CL-173) were purchased from the ATCC (Manassas, VA, USA) and were maintained at 37°C in an atmosphere of 5% CO_2_. Dulbecco’s modified Eagle’s medium (DMEM) or Roswell Park Memorial Institute (RPMI) 1640 medium (Lonza, Walkersville, MD, USA) containing 10% fetal bovine serum (FBS; Biotechnics Research, Lake Forest, CA, USA) and 1% penicillin/streptomycin (Cellgro, Manassas, VA, USA) was used for CT-26 and C2C12 cells or Raw 264.7 cells, respectively.

### Animals

Six-week-old male BALB/c mice were purchased from Taconic Farms (Samtako Bio Korea, Osan, Korea) and were housed under specific pathogen-free conditions (12 h/12 h light/dark cycle, 22 ± 1°C, 55 ± 5% humidity). Animal experiments were approved by the Animal Care and Use Committee of the Korea Institute of Oriental Medicine (KIOM, Daejeon, Korea; reference numbers #13-100, #14-074, and #15-011) and were carried out in accordance with the guidelines of the Animal Care and Use Committee of KIOM.

### Preparation of murine peritoneal macrophages

To prepare peritoneal macrophages, male BALB/c mice were each injected intraperitoneally with 300 μL sterile 3% sodium thioglycollate (Sigma Chemical Co., St. Louis, MO, USA). On day 3, macrophages were harvested by irrigating the abdominal cavity with 10 mL cold phosphate-buffered saline (PBS), and red blood cells (RBCs) were lysed with RBC lysis buffer, and then cells suspended in 10% FBS/RPMI medium were incubated in a 5% CO_2_ incubator at 37°C overnight. After adding fresh medium, the cells attached to the surface of the culture plate were used.

### Reagents and antibodies

Lipopolysaccharide (LPS) from *Escherichia coli* and recombinant murine tumor necrosis factor-α (rmTNF-α) were obtained from Sigma Chemical Co. and Promokine (Heidelberg, Germany), respectively. Ultra-pure bovine serum albumin (BSA) and Tween 20 were purchased from GenDEPOT Inc. (Barker, TX, USA) and AMRESCO (Solon, OH, USA), respectively. Antibodies against Bcl-2, XIAP, Bax, Bad, poly ADP ribose polymerase (PARP), inducible nitric oxide synthase (iNOS), p38, p-p38 (Thr180/Tyr182), extracellular regulated kinase (ERK)1/2, p-ERK1/2 (Thr202/Tyr204), c-jun N-terminal kinase (JNK), p-JNK (Thr183/Tyr185), AMP-activated protein kinase (AMPK), p-AMPK (Thr172), IκBα, p-IκBα (Ser32), and tubulin were purchased from Cell Signaling Technology (Danvers, MA, USA). An anti-myosin heavy chain (MyH) antibody and the ER Stress Antibody kit, including antibodies against Bip, ER oxidoreductase 1 (Ero1)-Lα, inositol-requiring enzyme (IRE)1α, protein disulfide isomerase (PDI), C/EBP homologous protein (CHOP), and protein kinase RNA-like ER kinase (PERK), were obtained from R&D Systems (Minneapolis, MN, USA) and Cell Signaling Technology, respectively. Dexamethasone and Compound C were purchased from Sigma Chemical Co., and mitogen-activated protein kinase (MAPK) inhibitors, including SP600125, SB203580, and PD98059, were purchased from Calbiochem (San Diego, CA, USA), respectively.

### Preparation of SGE

All dried herbs, including *Ginseng Radix alba, Atractylodis Rhizoma alba,* and *Hoelen*, were purchased from Yeoncheon Hyundai Herbal market (Yeoncheon, Korea), confirmed the identity by Professor Ki Hwan Bae (Chungnam National University, Korea), and stored in the herbal bank of the Korean Medicine Application Center (Daegu, Korea). The origin of each herb is listed in Supplementary Table 1. To prepare SGE, 16.67 g of each herb was ground into powder and then extracted in 500 mL 70% ethanol in a 37°C shaking incubator at 100 rpm for 24 h. The extract was filtered using 185 mm filter paper (Whatman, Piscataway, NJ, USA) and concentrated using a rotary vacuum evaporator (Buchi, Tokyo, Japan). After freeze-drying, the SGE powder was collected and weighed to 1.96 g; therefore, the yield was 3.92%. For *in vitro* experiments, SGE powder was dissolved in 10% DMSO (v/v) to a final concentration of 50 mg/mL, filtered through a 0.22-μm disk filter and then stored at −20°C until use.

### Cell viability assay and staining of live/dead cells

The effect of SGE on cell viability was determined using the Cell Counting Kit-8 (CCK, Donjindo Laboratories, Kumamoto, Japan) according to the manufacturer’s instructions. Briefly, cells were seeded in 96-well culture plates (0.5–1×10^4^/well/100 μL), allowed to attach to the plates, and then treated with varying concentrations of SGE for 24–48 h. CCK solution (10 μL/well) was added to each well, and the absorbance at 450 nm was measured using the SpectraMaxi3 microplate reader (Molecular Devices, Sunnyvale, CA, USA) after further incubation for 1 h. In addition, the live and dead cells were visualized using the LIVE/DEAD Cell Imaging Kit (Invitrogen, Carlsbad, CA, USA) according to the manufacturer’s protocol. Live (green) and dead (red) cells were observed under a fluorescence microscope (Nikon Eclipse Ti, Nikon instruments, Kanagawa, Japan).

### Western blot analysis

After washing the cells with PBS, cell lysates were prepared using the M-PER Mammalian Protein Extraction Reagent (Thermo Scientific, Rockford, IL, USA) according to the manufacturer’s instructions. The lysate protein concentration was determined using the Pierce^™^ Bicinchoninic Acid protein assay kit (Thermo Scientific), and equal amounts of each protein sample were mixed with NuPAGE 4× lithium dodecyl sulfate sample buffer (Invitrogen) and denatured by heating for 10 min at 95°C. The samples were separated by 8–15% SDS-PAGE and electro-transferred to a polyvinylidene difluoride membrane (Immobilon-P, Millipore, DARMSTADT, Germany). Membranes were blocked in a 3% BSA Tris-buffered saline solution containing 0.05% Tween 20 (TBST) for 1 h at room temperature and were incubated first with specific antibodies overnight at 4°C and then with HRP-conjugated secondary antibodies for 1 h at room temperature. After washing with TBST, immunoreactive bands were visualized using the Bio-Rad Clarity^™^ Western ECL Substrate under the ChemiDoc^™^ Touch Imaging System (Bio-Rad, Hercules, CA, USA). The band intensity was measured using ImageJ software (National Institute of Health, Bethesda, MD, USA).

### Reverse transcription-polymerase chain reaction (RT-PCR)

Total RNA was extracted using an RNA extraction solution (BioAssay Co., Daejeon, Korea) and reverse transcribed using the 1^st^ Strand cDNA Synthesis kit (BioAssay Co.) according to the manufacturer’s protocol. cDNA amplification of iNOS, IL-6, TNF-α, and IL-1α was performed in the Vertiti 96-well Thermal Cycler (Applied Biosystems, Foster City, CA, USA), and the DNA products were visualized on a 1% agarose gel by staining with GreenLight^™^ (BioAssay Co.). The relative band intensity was calculated using ImageJ software.

### Determination of NO levels in culture supernatants

Cells were pre-treated with the indicated concentrations of SGE for 1 h and then stimulated with LPS (200 ng/mL) for 24 h. Culture supernatants were obtained after centrifugation at 12,000 rpm for 10 min to remove cell debris and were mixed with the same volume of Griess reagent (1% sulfanilamide, 0.1% naphthylethylenediamine dihydrochloride, and 2.5% phosphoric acid). After incubation for 5 min at room temperature, the absorbance at 570 nm was measured using the SpectraMaxi3 Multi-mode reader (Molecular Devices).

### Cytokine enzyme-linked immunosorbent assay (ELISA)

The protein levels of mouse IL-1β, IL-6, and TNF-α in culture supernatants and mouse sera were evaluated using the ELISA-Ready-SET-Go kit (eBioscience, San Diego, CA, USA) according to the manufacturer’s protocol.

### Preparation of CT-26-conditioned medium (CM)

CT-26 cells suspended in 10% FBS/DMEM were seeded in 100 mm culture dishes at a density of 5×10^4^ cells/cm^2^, allowed to adhere, and then treated with the indicated concentrations of SGE. After 24 h, the cells were washed three times with PBS, additionally washed twice with serum-free DMEM, and then incubated for 24 h in serum-free DMEM. The CM was collected and centrifuged to remove cell debris followed by filtration using a 0.22 μm disk filter.

### Detection of C2C12 myoblast proliferation, myotube differentiation, and myotube degradation

CT-26 CM was diluted 1:5 with either 10% FBS/DMEM (growth medium; GM) or 5% horse serum (HS; Gibco-BRL, Grand Island, NY, USA)/DMEM (differentiation medium; DM), and the appropriate quantities of FBS, HS, and antibiotics were added to compensate for CM. To measure C2C12 myoblast proliferation, cells seeded in the 96-well culture plate (1×10^3^ cells/well) were treated with control GM, SGE-treated CT-26 CM, or untreated CT-26 CM. After incubation for 48 h at 37°C, cell proliferation was observed and measured using the CCK-8 kit. To induce myogenic differentiation, C2C12 myoblasts at approximately 80% confluency were incubated in control DM, SGE-treated CT-26 CM, or untreated CT-26 CM for 3–7 days. Degradation of the myotube was induced by addition of CT-26 CM or TNF-α (20 ng/mL).

### Measurement of lipid accumulation in 3T3-L1 adipocytes

To induce adipogenic differentiation, 3T3-L1 cells cultured to confluency in 10% FBS/DMEM were incubated in adipocyte DM consisting of 10% FBS/DMEM, insulin (1 μg/mL), dexamethasone (1 μM), and 3-isobutyl-1-methylxanthine (0.5 mM) for 3 days. Thereafter, cells were maintained in 10% FBS/DMEM containing insulin (10 μg/mL) for 2 days and in 10% FBS/DMEM for another 2 days, changing the medium daily. In tumor-induced lipolysis, mature 3T3-L1 adipocytes were incubated with SGE-treated or untreated CT-26 CM for 2 days, and then the cellular accumulation of neutral lipid vacuoles was assessed by Oil Red O staining. The retained red dye was eluted by isopropanol, and the absorbance was measured at 520 nm.

### Experiments for *in vivo* cancer-induced cachexia

To induce cancer-induced cachexia, CT-26 cells (3×10^6^ cells/mouse) were subcutaneously injected into the abdominal region of male BALB/c mice. On day 5 after tumor injection, the body weight and food intake were decreased in tumor-bearing mice by approximately 10% compared with that in normal mice (no tumor + saline). Tumor-bearing mice were divided randomly into three groups and orally administered saline (tumor + saline; control mice) or SGE daily at doses of 10 and 50 mg/kg in a volume of 100 μL for 15 days. Healthy normal mice were also administered an equal volume of saline. During the experiment, the body weight, food intake, and tumor volume were measured every 3 days. On day 20 after tumor injection, tumors, epididymal fat, abdominal subcutaneous fat, gastrocnemius muscle, and the heart were resected from mice after sacrifice by CO_2_ inhalation and then weighed. In addition, whole blood samples were collected, and the IL-6 levels in sera were determined by ELISA. To measure the carcass weight, blood was exsanguinated, and the remaining viscera were clearly wiped out using a gauze pad.

### Statistics

The data are presented as means ± standard deviation (SD). Significantly different mean values between the two groups were analyzed by Student’s *t*-test. The treatment effect was analyzed using one-way ANOVA by Dunnett’s multiple comparison test. A *p*-value less than 0.05 was deemed to indicate statistical significance. All the variables were analyzed using GraphPad Prism5 (GraphPad software Inc., La Jolla, CA, USA).

## SUPPLEMENTARY MATERIALS FIGURES AND TABLES


