# Supplementary material for: A novel herbal formula, SGE, induces endoplasmic reticulum stress-mediated cancer cell death and alleviates cachexia symptoms induced by colon-26 adenocarcinoma
Source: Oncotarget. 2018 Mar 27;9(23):16284–96. doi: 10.18632/oncotarget.24616 (PMC5893240; doi:10.18632/oncotarget.24616)
Supplement: Supplementary file 1 [file oncotarget-09-16284-s001.pdf]

A novel herbal formula, SGE, induces endoplasmic reticulum stress-mediated cancer cell death and alleviates cachexia symptoms induced by colon-26 adenocarcinoma

SUPPLEMENTARY MATERIALS

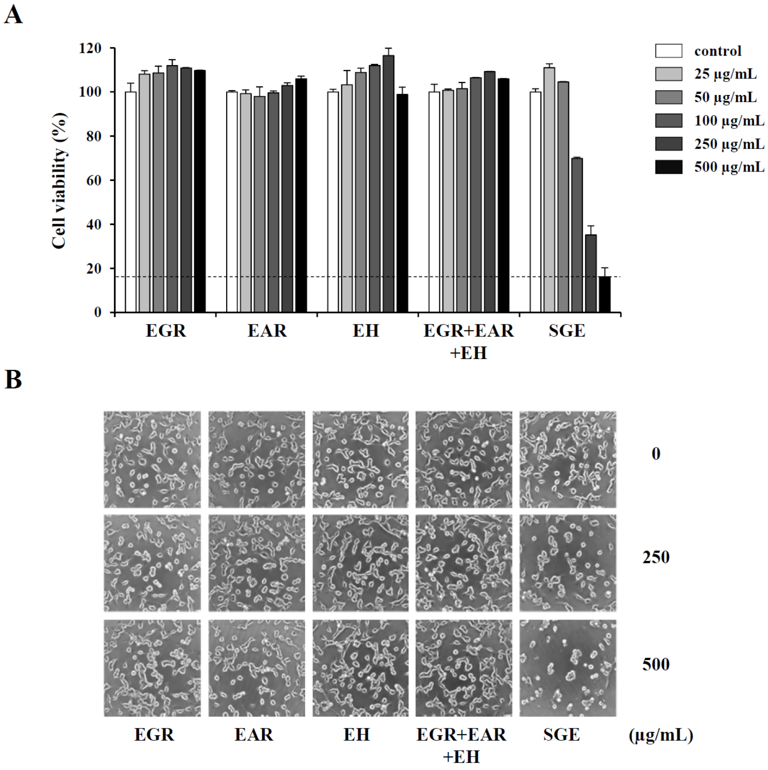

Supplementary Figure 1:SGE as a cocktail induces cell death in CT-26 murine colon carcinoma cells. **(A)** CT-26 cells were incubated with the indicated doses of SGE, single medicinal herb of SGE such as EGR, EAR, and EH, or their co-treatment. After 48 h, cell viability was determined using the CCK assay and expressed as the mean ± standard deviation (SD). **(B)** Morphological change in CT-26 cells treated as indicated was observed under an inverted microscope at a magnification of × 200.

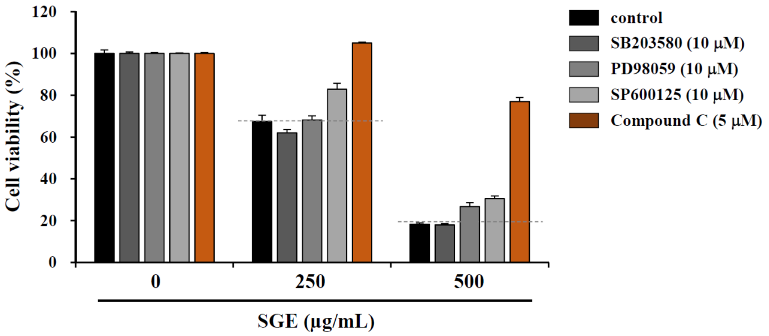

Supplementary Figure 2: AMPK activation is critical for the SGE-induced cell death in CT-26 murine colon carcinoma cells. Cells pretreated with or without specific pharmacological inhibitors for p38 (SB203580, 10 µM), ERK (PD98059, 10 µM), JNK (SP600125, 10 µM), and AMPK (Compound C, 5 µM) for 1 h were treated with 250 and 500 µg/mL SGE. After incubation for 24 h, cell viability was assessed by CCK assay and expressed as the mean ± SD.

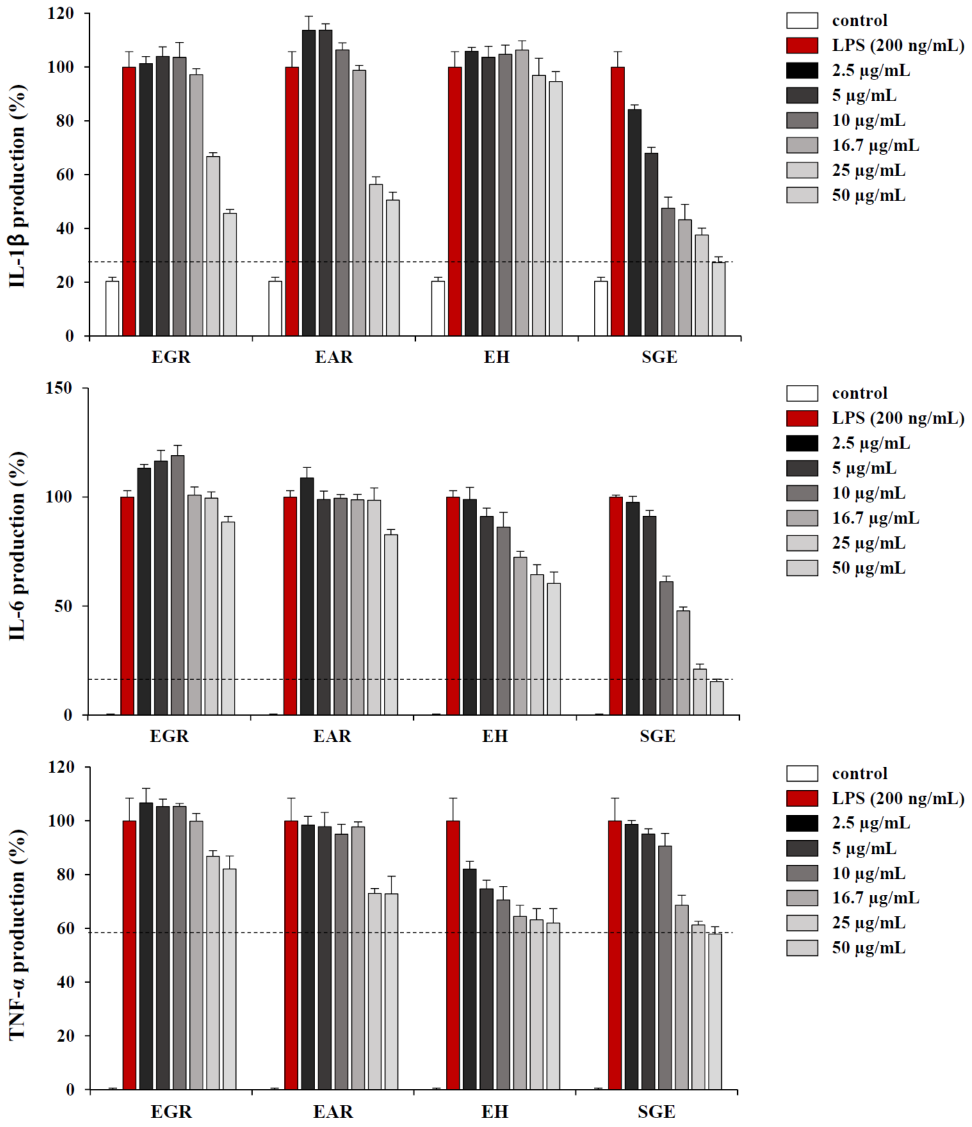

Supplementary Figure 3: SGE as a cocktail remarkably suppresses LPS-induced production of inflammatory cytokines in Raw 264.7 cells. Raw 264.7 cells were pretreated with or without the indicated doses of SGE or single medicinal herb of SGE such as EGR, EAR, and EH for 1 h, and stimulated with LPS (200 ng/mL) for 24 h. After collecting the culture supernatants, the levels of IL-1β, IL-6, and TNF-α were measured by ELISA. Data are expressed as the mean ± SD performed in triplicate**.**

Supplementary Table 1: Origin of each herb in SGE

Latin name	Scientific name	Origin
Ginseng Radix alba	Panax ginseng C. A. Meyer	Geumsan, Korea
Atractylodis Rhizoma alba	Atractylodes japonica Koidzumi	Yeongcheon, Korea
Hoelen	Wolfiporia extensa	Yeongwol, Korea

Supplementary Table 2: Body weight of mice administered with SGE or saline

Treatment	Body weight (g)				
	Day 0	Day 3	Day 7	Day 10	Day 14
Saline	20.41 ± 1.32	21.61 ± 1.18	22.28 ± 1.69	22.83 ± 1.73	23.49 ± 1.48
100 mg/kg	20.48 ± 0.11	21.60 ± 0.23	22.27 ± 0.18	22.91 ± 0.21	23.67 ± 0.62

Each group of male BALB/c mice (n=5) were orally administered with 100 mg/kg SGE or same volume of saline daily for 14 days. Body weights were weighed at 0, 3, 7, 10, and 14 days. Data are expressed as mean ± S.D.

Supplementary Table 3: Organ weight of mice administered with SGE or saline

Treatment	Organ weight (g)					
	Lung	Liver	Heart	Spleen	Kidney (L)	Kidney (R)
Saline	0.17 ± 0.01	1.44 ± 0.20	0.11 ± 0.01	0.08 ± 0.01	0.19 ± 0.02	0.19 ± 0.00
100 mg/kg	0.18 ± 0.00	1.44 ± 0.08	0.12 ± 0.01	0.08 ± 0.00	0.19 ± 0.00	0.18 ± 0.00

Each group of male BALB/c mice (n=5) were orally administered with 100 mg/kg SGE or same volume of saline daily for 14 days. At day 14, mice were sacrificed and organs were weighed. Data are expressed as mean ± S.D.

Supplementary Table 4: Assessment of safety in mice administered with SGE or saline

Treatment	Serum analysis			
	GOT (IU/L)	GPT (IU/L)	BUN (mg/dL)	CRE (mg/dL)
Saline	60.45 ± 1.41	43.25 ± 0.71	25.83 ± 1.84	0.45 ± 0.07
100 mg/kg	53.55 ± 0.71	41.55 ± 7.07	25.80 ± 3.39	0.45 ± 0.07

Each group of male BALB/c mice (n=5) were orally administered with 100 mg/kg SGE or same volume of saline daily for 14 days. At day 14, mice were sacrificed and the levels of GOT, GPT, BUN, and CRE were analyzed; GOT, glutamic oxaloacetic transaminase; GPT, glutamic pyruvic transaminase; BUN, blood urea nitrogen; CRE, creatinine. Data are presented as means ± S.D.
